# Non-communicable Disease Risk Reduction Teaching in India: A Curricular Landscape

**DOI:** 10.3389/fpubh.2019.00133

**Published:** 2019-06-04

**Authors:** Sanghamitra Pati, Rajeshwari Sinha, Pranab Mahapatra

**Affiliations:** ^1^Department of Health Research, ICMR Regional Medical Research Centre, Bhubaneswar, India; ^2^Independent Researcher, New Delhi, India; ^3^Department of Psychiatry, Kalinga Institute of Medical Sciences, Bhubaneswar, India

**Keywords:** non-communicable diseases, NCD risk factor, smoking cessation, alcohol consumption, obesity, physical activity, diet

## Abstract

Non-communicable diseases (NCDs) have today become a growing epidemic, surpassing infectious diseases and adversely impacting national health systems, policies, and socio-economic developments. Management of NCD and its risk factors such as tobacco use, poor diet, physical inactivity, and obesity has garnered paramount importance under the Sustainable Development Goals (SDGs). However, the educational settings for healthcare professionals to prepare them for delivery of preventive interventions w.r.t. to NCD risk factors require a stronger foundation. In the present work, a landscaping exercise of the teaching of NCD risk factors in the extant Bachelor of Medicine and Bachelor of Surgery (MBBS) curricula of healthcare professionals from select medical colleges in India has been undertaken. Our assessment indicates that in the present MBBS curriculum, effort is largely directed upon teaching only specific aspects of NCDs, such as hypertension, diabetes, mental illness, blindness, and cancer with greater inclination toward clinical aspects. With regard to NCD risk factors, there is inadequate focus on the healthcare promotion aspects. The overall syllabus also does not address long-term health implications of tobacco smoking, lack of proper diet or physical activity, excessive alcohol consumption etc. It also does not reflect any practical trainings or sessions related to health promotion or behavior change activities. As a way forward, we suggest that NCD risk reduction aspects be incorporated extensively into existing health professional education through the development of a curricular framework and an intervention plan contextualized to India. Findings from this study are expected to help provide critical inputs for the design and development of a curricular plan with thrust on tobacco cessation, physical activity, healthy diet, and restricted alcohol consumption, leading to creation of a health competent workforce engaged in NCD prevention and health promotion.

## Introduction

Non-communicable diseases (NCDs) or chronic diseases have today surpassed infectious diseases as the major cause of mortality and morbidity. As per the World Health Organization (WHO), 71% of global deaths in 2016 were attributed to NCDs (1). In India, NCDs have emerged as the leading causes of death, accounting for 61% of total deaths (2). This growing epidemic has been severely impacting national health systems, policies, and socio-economic developments. The WHO has recently recognized NCDs as one among the top 10 global health threats of 2019.

The primary contributors to NCD are cardiovascular diseases (CVDs), cancer, chronic respiratory disorders, and diabetes. Much of the burden of these NCDs can be attributed to lifestyle-related risk factors such as tobacco use, physical inactivity, alcohol consumption, and obesity. Direct or indirect exposure to tobacco not only increases the risk of premature deaths from NCDs, but also imposes additional economic burden and reduced productivity. Recent estimates indicate that tobacco use has led to 6 million preventable deaths per year globally (3). Similarly, excessive consumption of alcohol has been linked to the risk of developing mental and behavioral disorders. Insufficient physical activity is another factor responsible for growing obesity, raised blood pressure, or hypertension in individuals. Both physical inactivity and alcohol consumption are burgeoning in proportion to the economic progress in countries.

Given the strong linkages between risk factors and NCDs, their management assumes paramount importance. One of the ways in which this could be achieved is by ensuring increased health education and promotion among healthcare providers. The importance of a physicians' role in improving the health status of the general population is well-recognized. However, a major challenge is to have a critical mass of human resources trained in the specific delivery of healthcare. Studies have shown that healthcare providers do not feel competent enough to address healthcare-related issues in their daily practice, including those related to NCDs (4). They often feel that the education received in their formative phase has not prepared them effectively for executing such a role on the ground.

In the context of NCD care, knowledge both on healthcare delivery on the ground as well as healthcare promotion to reduce exposure to major risk factors is critical. It is therefore essential that healthcare educational settings encourage appropriate capacity development w.r.t. NCD care, right from the formative stages of education. Several international literatures have documented the lack of focus on NCD risk factor education in medical education (5–8). In order to understand where we stand on NCD risk factor prevention and control in medical education in India, the present work was undertaken. Since undergraduate education forms the basis of knowledge development in any particular domain, a landscaping of the extant Bachelor of Medicine and Bachelor of Surgery (MBBS) curricula in the country has been carried out. Content specific to NCD risk factors in the MBBS syllabus of select universities/autonomous institutions offering medical education across India was mapped and analyzed. The observations reflect the Indian scenario and provide inputs toward the need for curricular interventions w.r.t. tobacco cessation, physical activity, healthy diet, and restricted alcohol consumption. We expect that the findings would also be helpful in curricular integration to enable containment of NCD and their risk factors.

## Methodology

The study was conducted between September and December, 2018. It involved an analysis of NCD risk reduction–related teaching content within the extant curricula of undergraduate medical (MBBS) syllabus in India. A systematic, predetermined strategy was undertaken for collecting and collating the required information. The methodology used was similar to that adopted in an earlier study involving following process (9, 10).

A thorough internet search was carried out to collect information in the public domain regarding MBBS courses offered across medical universities or autonomous medical institutions in India. The search was conducted using search engines like Google and Dogpile. A set of key words was used for the search which included MBBS, medical education, syllabus, medical universities in India, and universities offering medical education in India. Once a list of medical universities or institutions offering MBBS education was shortlisted, the websites of each of them were visited and detailed information on undergraduate MBBS syllabus collected. The search was limited to full-time MBBS courses offered only in India. Universities/autonomous institutions for which no information on syllabus was available on website were excluded. Similarly, short-term courses offered by various institutions, lasting from a few days to a few weeks, were not taken into consideration. Overall, eight medical universities and institutions were shortlisted. In parallel, the websites of Medical Council of India (MCI), Nursing Council of India, and Ministry of Health and Family Welfare (MOHFW) were also referred to for additional information, if any. The recently released curriculum by the MCI, which is followed by a large number of universities across India, was also reviewed.

Each syllabus was thoroughly assessed for understanding the following: (i) broad contents, (ii) whether NCD is a part of the undergraduate syllabus or teaching curriculum, and (iii) whether NCD risk factor reduction is a part of the undergraduate syllabus or teaching curriculum, and the observations were further analyzed.

## Results

A landscaping of the NCD-related content in the extant MBBS curricula in India that is available in public domain has been carried out. Table 1 provides a summary of the various topics in NCDs or its risk factors within the MBBS curriculum followed by selected eight medical universities/autonomous institutions. Colleges belonging to these eight universities/autonomous institutions were largely MCI recognized with few exceptions.

**Table 1:** Summary of non-communicable disease (NCD) and NCD risk factor–related teaching content in Bachelor of Medicine and Bachelor of Surgery (MBBS) syllabus of select medical universities/autonomous institutions in India.

**Main topic**	**Sub-topic**	**Specific/key areas covered**	**Reference(s)**
**ALL INDIA INSTITUTE OF MEDICAL SCIENCES, NEW DELHI**			
Community medicine	Epidemiology	Natural history of NCDs, principles of NCD control	(11)
	Epidemiology of communicable diseases and NCDs	Hypertension, RHD, CHD, cancers, blindness, road traffic accidents, diabetes mellitus, obesity, nutritional disorders	
	Nutrition	Role of nutrition in health and disease, nutritional requirements and sources, balanced diet, nutritional programs	
Psychiatry	Alcohol dependence	Concepts of alcohol abuse and dependence, epidemiology of alcohol dependence; clinical features, withdrawal symptoms, etiology, outcome, treatment	
**PUNJAB UNIVERSITY, CHANDIGARH**			
Biochemistry	Energy and nutrition	Balanced and adequate diets, formulation of diets in health and diseases, obesity, starvation	(12)
Pathology	Central nervous system and special senses	Effects of aliphatic alcohols on different organ systems; acute and chronic alcoholism, methyl alcohol poisoning	
	Cardiovascular system	Hypertension: definition, classification, pathogenesis, morphological changes	
Community medicine	Nutrition	Nutritional requirements, balanced diet, common deficiency diseases, assessment of nutritional status; national nutritional health policy and programs	
Specific epidemiology	NCDs	Epidemiology and prevention of CHD, RHD, hypertension, cancers, diabetes, blindness; accidents; smoking and alcohol in health and disease, public health importance of a particular disease, control of NCDs	
Medicine	Nutrition	Obesity: definition, BMI, etiology, pathophysiology, clinical features, complications, treatmentAlcohol: measures, metabolism, problems in chronic alcoholics and binge drinkers, physical effects of excess alcohol consumption, dependence, withdrawal, alcohol poisoning	
	Psychiatry	Alcoholism epidemiology, etiology, detection of heavy drinkers; clinical and laboratory diagnosis of excess alcohol intake; dependence, withdrawal symptoms, treatment	
**KRISHNA INSTITUTE OF MEDICAL SCIENCES UNIVERSITY, MAHARASHTRA**			
Human biochemistry	Nutrition	Balanced diet	(13)
Pathology	Deficiency disorders	Obesity	
	Alcoholic liver disease and cirrhosis	Pathogenesis, morphological manifestations and correlation with clinical features of alcoholic liver disease and cirrhosis	
	RHD and CHD	Incidence, etiopathogenesis, morbid anatomy, histopathology, clinical course etc.	
Pharmacology and pharmacotherapeutics	Cardiovascular system	Drug therapy of hypertension	
Psychiatry		Alcohol dependence	
Medicine and allied subjects	Nutrition	Balanced diet, obesity diagnosis, complications, and management	
	Cardiovascular system	CHD: etiology and classification, CHD in adults and its importance, hypertension	
Community medicine including humanities	Community medicine	Epidemiology of NCDs; introduction to NCDs: RHD, cancer, obesity/diabetes (practicals)	
**INTEGRAL UNIVERSITY, UTTAR PRADESH**			
Community medicine	Epidemiology of communicable diseases and NCDs	NCDs: nutritional disorders, RHD, CHD, hypertension, cancers, blindness, road traffic accidents, diabetes mellitus, obesity	(14)
Medicine	Nutrition and health	Nutritional assessment needs, nutrition and metabolic disorders, obesity	
**DR. NTR UNIVERSITY OF HEALTH SCIENCES, ANDHRA PRADESH**			
Biochemistry	Nutrition	Balance diet, nutritional disorders	(15)
Pharmacology		Hazards of smoking, alcohol, narcotics	
Community medicine		NCD: diabetes, hypertension, heart diseases, blindness, accidents, geriatric problems	
**RAJIV GANDHI UNIVERSITY OF HEALTH SCIENCES, KARNATAKA**			
Pathology	Cardiovascular pathology	RHD, hypertension, and hypertensive heart disease	(16)
Community medicine	Nutrition and dietetics	Common nutritional disorders and deficiencies, national nutrition programs	
	Epidemiology of communicable and non-communicable diseases	NCDs: CHD, hypertension, RHD, cancers, diabetes, blindness, accidents	
Psychiatry	Nutrition/exposure to physical and chemical agents	Clinical features, diagnosis and management of alcoholism, drug abuse	
Medicine and its allied specialties	Nutrition/exposure to physical and chemical agents	Acute and chronic effects of alcohol and their management, nutrition and dietary management (e.g., obesity)	
	Cardiovascular system	Hypertension and hypertensive heart disease, RHD	
	Gastrointestinal tract	Cirrhosis of liver	
**PONDICHERRY UNIVERSITY, PUDUCHERRY**			
Biochemistry	Nutrition	Balanced and adequate diets, formulation of diets in health and diseases, obesity	(17)
Community medicine	Epidemiology of specific diseases	Epidemiology of NCDs, their control, prevention	
General medicine	Nutrition and nutritional disorders	Obesity, diet therapy	
Psychiatry and behavioral sciences	Psychiatry and drug/alcohol de-addiction	Alcohol use, problem drinking, education, and awareness; signs and symptoms of alcoholism, its medical and psychosocial impact, treatments available	
**JAWAHARLAL INSTITUTE OF POSTGRADUATE MEDICAL EDUCATION AND RESEARCH, PUDUCHERRY**			
Cardiovascular system		Blood pressure (mechanism and regulation), hypertension	(18)
Preventive and social medicine	Nutrition	Balanced diet, dietary goals, nutritional deficiency	
	NCD epidemiology	Introduction to NCD and mental health, risk factors for NCD, diabetes mellitus, cardiovascular diseases, cancers, blindness, road-traffic injuries	
Pathology	Cell injury, inflammation and repair, hemodynamics, diseases of immune system, genetic and metabolic diseases, neoplasia, and nutritional diseases	Nutritional diseases: obesity Neoplasia: Smoking and cancer	
	Hematology, reticulo-endothelial system, respiratory system, and environmental diseases	Hazards of smoking, alcohol and radiation, deleterious effects of tobacco, deleterious effects of alcohol	
	Cardiovascular system, gastrointestinal system, and hepatobiliary including pancreas	Cardiovascular system: hypertension, RHD, IHD Diseases of liver and gall bladder: cirrhosis and its etiology, classification, morphology, complications; alcoholic liver disease	
Forensic medicine	Transportation injuries	Alcohol, drugs, and trauma	
	Inebriants—alcohol	Signs and symptoms (acute poisoning), treatment, chronic alcoholism, alcoholic hallucinosis, drunkenness, and its diagnosis	

*RHD, rheumatic heart disease; CHD, coronary heart disease; IHD, ischemic heart disease; BMI, body mass index*.

An analysis of Table 1 indicates that present effort is largely directed upon teaching only specific aspects of NCDs such as hypertension, diabetes, mental illness, blindness, and cancer, with greater inclination toward clinical aspects, epidemiology, pathogenesis, examination, and management etc. There are no focus on management of factors that give rise to NCDs. There is also focus on teaching various aspects of nutrition such as balanced diet and malnutrition effects.

With regard to NCD risk factors such as tobacco or smoking cessation, physical activity, and reduced alcohol consumption, there appears a gap with regard to teaching. Although some of the syllabi examined do refer to teaching on alcohol dependence, alcohol de-addiction, hazards of smoking, or alcohol consumption, these are largely inclined toward their clinical aspects such as metabolism and epidemiology. There is little mention about healthcare promotion aspects, i.e., what is to be done for prevention or control of these risk factors. Moreover, contents related to smoking or alcohol consumption are placed in a fragmented manner in the syllabi. For example, while alcohol de-addiction forms a part of psychiatry component in some syllabus, it is also covered under pharmacology, pathology, or nutrition segments in others. The effects of acute and chronic alcoholism and alcohol poisoning have been attributed to pathology division in some syllabus. The landscaping also indicates limited attention on role of regular exercise or physical activity.

The overall syllabus also does not address topics such as long-term implications of tobacco smoking on chronic ailments; need for cessation or counseling to limit tobacco smoking; steps to be able to manage relapse cases; linkages of physical activity with reduced obesity, hypertension, diabetes etc.; and benefits of reduced alcohol dependence on mental health etc. All these are not mandated as part of the formal medical curriculum and are possibly left to the discretion of the faculty who is teaching the course. The revised MBBS syllabus of the Jawaharlal Institute of Postgraduate Medical Education and Research, however, focuses on elements such as hazards of smoking and alcohol consumption and deleterious effects of tobacco and alcohol. Moreover, the present curriculum also does not reflect any practical trainings or sessions related to such health promotion or behavior change activities.

There are about 494 medical colleges that offer MBBS education in India, nearly 75% of which are presently recognized by the MCI (19). Very recently, the MCI released a revised set of competency based curriculum, which will be implemented from August 2019 for first-year MBBS students this year. Interestingly, this revised curriculum stresses on skills and competencies of healthcare professionals such that they can be trained to provide preventative, promotive, curative, palliative, and holistic care. The new curriculum pays attention to the need for addressing NCD risk factors and designing strategies for their prevention. For example, it focuses on understanding risk factors of obesity, importance of communication and patient counseling on issues such as smoking cessation, and importance of lifestyle modification such as reduced alcohol intake and increased exercise. This is a noteworthy upgradation that MCI has brought about in view of creating a mass of skilled professionals. The revised curriculum also reflects efforts toward reducing compartmentalization of disciplines and promoting horizontal and vertical integration in the medical curriculum for enriched learning among students. Implementation of this curriculum is likely to bring a major shift in NCD risk factor teaching in India.

The above observations pertain to those obtained from curricular mapping. However, all the components as per syllabus may not get implemented in classroom settings, owing to context specific issues. Similarly, NCD risk factor–related topics which may get transacted in class are not captured in the syllabus. Future studies exploring factual learning from classroom sessions should be undertaken to have a better clarity of the scenario.

## Discussion

### Present Status of Health Promotion Education w.r.t. NCD Risk Factors in Medical Curriculum

Health promotion entails a holistic approach of promoting health intervention to encourage better health and well-being. Inclusion of health promotion practices in routine care is imperative for a stronger healthcare system. It should be incorporated as a structured module in medical curriculum. In fact, the role of health promotion and education for improving lifestyle modification behaviors, such as smoking cessation and increasing physical activity that ultimately contribute to NCD management, has also been emphasized (20, 21). In this section, we discuss curricular interventions that have been introduced to bring down tobacco use, physical inactivity, or alcohol consumption. While all NCD risk factors have not been addressed altogether in any single curriculum, country-level efforts focused on individual risk factors have been documented.

Smoking and other tobacco exposure have been recognized as the most significant preventable factors in premature NCD morbidity and mortality. Cessation programs are essential components of comprehensive tobacco control. Healthcare providers, especially physicians, have major responsibility for promoting cessation, for which necessary skills and training are needed to be imparted among healthcare providers. Data available from India indicates the lack of skills in delivering brief intervention and counseling in tobacco cessation for physicians. Most physicians and allied healthcare professionals believe that they should address the issue of tobacco with their patients but are rarely provided with adequate training or support to do so effectively (22). Evidence from earlier Indian studies on the student's beliefs and practices of health promotion reported that even they did not feel well-prepared and competent enough to counsel patients about health issues (10). Very much like in the present case, international studies have also highlighted the inadequate focus on tobacco cessation in medical curriculum. A study based on curricula of medical schools in United Kingdom (UK) reflected that there was no mention of smoking or smoking cessation in the published curriculum material of 42% of medical schools, which the study had surveyed. It was thus concluded that teaching on smoking cessation in UK medical schools is inadequate (5). Another survey conducted across 33 medical schools in UK showed significant deficits in undergraduate medical training on smoking cessation (23). However, of the 22 schools which responded to this survey, 100, 95.5, and 22.7% reported that they imparted knowledge on linkages between smoking and cancer, CVDs, and mental health, respectively. Recognizing the critical role that physicians and other healthcare professionals play in reducing tobacco use, the World Health Organization's Tobacco Free Initiative and healthcare professional organizations from around the world have called for professions to encourage cessation among their patients (24). The Global Health Professional Student Survey (GHPSS) have also suggested introducing a separate integrated tobacco module into medical school curricula (25, 26).

Physical activity, proper diet, and nutrition are other key components of NCD prevention, which was found to be equally inadequately addressed in medical curriculum. For example, physical activity teaching content in the curricula of all medical schools in the UK was indicated to be sparse or non-existent overall (6). A similar scenario was observed in the United States (US) as well. A review of the amount and type of physical activity education in medical education in the US showed that majority of institutions did not offer any physical activity education–related courses (7). The study concluded that more than 50% of the physicians trained in the US in 2013 had received no formal education in physical activity and may, therefore, be not well-prepared to assist patients. On the other hand, medical schools in Australia consider it as their responsibility to impart physical activity training to their medical students. An evaluation study of the medical school curricula of Australia showed that 88.2% of the participating schools provided specific physical activity training to medical students (27). In addition, Australian medical education programs allocated much higher time for such training as compared to UK or US, thereby allowing more time for both teaching as well as preventative training.

Although the relevance of educational interventions and teaching medical students about alcohol is well-recognized, there is limited evidence for educators involved in building curriculum around this aspect. Despite an increase in prevalence of the problem of drug and alcohol use in Ireland and the UK, this has not been reflected in undergraduate medical curricula. Formal undergraduate teaching in the area of drug and alcohol misuse in the UK and Ireland is minimal (8). A 2014 report on undergraduate training in addiction medicine in Swiss universities showed that training lacked a clear teaching concept and was patchy and non-coordinated (28).

### Need for Incorporation of Health Promotion Aspects in Medical Curriculum

An understanding of the existing landscape w.r.t. NCD risk factor teaching in undergraduate medical curriculum indicates that it is essential to strengthen the same. This could be achieved through the development of an integrated health education program. A well-structured health promotion module that outlines the need for teaching on topics, such as physical activity and its linkages to obesity and weight management or need for smoking cessation counseling to patients, needs to be developed and incorporated in undergraduate curriculum.

It has also been observed that health promotion has never been incorporated in the duties or job responsibility of physician during primary care delivery services in India. This could result in “lay away” of health promotion practice compared to regular curative practices. In-service physicians should therefore be provided with hands-on training as part of their continuing medical education. Patient counseling on preventive aspects of NCD risk factors could be introduced as part of job responsibilities of primary care physicians as well. Moreover, raising awareness among patients and educating them on the risks factors of NCDs through necessary initiatives will be equally important for prevention and control of NCDs.

### Need for Integration in the Medical Curriculum

Moving beyond the traditional teaching methods, a comprehensive and integrated approach toward teaching is the need of the hour. This would not only help students correlate learning's from basic subjects in pre-clinical and para-clinical phases, but also understand their relevance while practicing medicine at the community level. Under NCD and NCD risk factors teaching, both horizontal and vertical integration will be important. The vertical integration of various aspects would converge present teaching efforts spread out under different departments or themes (as observed in our present analysis) under one topic. The student will therefore be able to conceptualize better and appreciate the nuances of dealing with an NCD or its risk factor, which has wide repercussions across organ systems. The revised MCI curriculum is such a step ahead in this direction.

## Way Forward

Prevention of NCDs, especially their risk factors, does not appear to feature prominently in medical school curriculum. Established risk factors which do find mention in some of the present curricula is present in a less integrated manner. In view of the present work, a way forward has been suggested below. It is envisaged that this would be relevant not only for India, but also for other countries wherein risk factor–related NCD education in undergraduate medical curriculum is limited.

- Incorporation of NCD risk reduction into existing health professional education. Greater focus on NCD risk reduction teaching coupled with strategic training can help build up a cadre of healthcare personnel who are competent in NCD prevention and management.
- Development of a curricular framework contextualized to the country setting. This could involve identification of necessary core competencies, understanding of global best practices, adaptation of globally accepted teaching models on smoking cessation, physical activity, diet, and nutrition etc.
- Development of a curricular intervention plan for incorporating the core competencies appropriate for the country. This could then be followed by design of strategies and techniques pertaining to how and when these topics could be introduced or integrated into both preclinical and clinical years.

## Author Contributions

SP conceptualized the work. RS and SP worked on the research, data collection, data analysis, and drafting of the manuscript. SP and PM reviewed the manuscript.

### Conflict of Interest Statement

The authors declare that the research was conducted in the absence of any commercial or financial relationships that could be construed as a potential conflict of interest.

## References

[1] World Health Organization Global Health Observatory Data: Deaths From NCDs. Available online at: https://www.who.int/gho/ncd/mortality_morbidity/en/

[2] World Health Organization Non-communicable Diseases Progress Monitor. (2017). Available online at: http://apps.who.int/iris/bitstream/handle/10665/258940/9789241513029-eng.pdf?sequence = 1

[3] World Health Organization Global status report on noncommunicable diseases 2014. (2014). Available online at: https://www.who.int/nmh/publications/ncd-status-report-2014/en/

[4] Pati S Chauhan AS Mahapatra S Sinha R Pati S. Practicing health promotion in primary care–a reflective enquiry. J Prev Med Hyg. (2017) 58:E288–93. 10.15167/2421-4248/jpmh2017.58.4.749 29707659 PMC5912790

[5] Roddy E Rubin P Britton J. A study of smoking and smoking cessation on the curricula of UK medical schools. Tob Control. (2004) 13:74–77. 10.1136/tc.2003.004572 14985601 PMC1747835

[6] Weiler R Chew S Coombs N Hamer M Stamatakis E. Physical activity education in the undergraduate curricula of all UK medical schools. Are tomorrow's doctors equipped to follow clinical guidelines? Br J Sports Med. (2012) 46:1024–6. 10.1136/bjsports-2012-091380 22846233 PMC3856633

[7] Cardinal BJ Park EA Kim M Cardinal MK. If exercise is medicine, where is exercise in medicine? Review of US medical education curricula for physical activity-related content. J Phys Act Health. (2015) 12:1336–43. 10.1123/jpah.2014-0316 25459966

[8] O'Brien S Cullen W. Undergraduate medical education in substance use in Ireland: a review of the literature and discussion paper. Ir J Med Sci. (2011) 180:787. 10.1007/s11845-011-0736-y 21805088

[9] Zodpey SP Negandhi H Tiwari RR. Mapping ‘Occupational Health’ courses in India: a systematic review. Indian J Occup Environ Med. (2009) 13:135–40. 10.4103/0019-5278.58917 20442832 PMC2862446

[10] Pati S Sharma K Zodpey S Chauhan K Dobe M. Health promotion education in India: present landscape and future vistas. Glob J Health Sci. (2012) 4:159–67. 10.5539/gjhs.v4n4p159 22980352 PMC4776916

[11] All India Institute of Medical Sciences. Syllabus MBBS at the AIIMS. Available online at: https://www.aiims.edu/aiims/academic/aiims-syllabus/Syllabus%20-%20MBBS.pdf

[12] Punjab University Chandigarh Faculty of Medical Sciences: Syllabi for Bachelor of Medicine and Bachelor of Surgery (M.B.B.S) for the Admission Batch 2012. Available online at: http://puchd.ac.in/includes/syllabus/2012/20120718152909-M.B.B.S,-2012.pdf?201912151801

[13] Krishna Institute of Medical Sciences Syllabus and Examination pattern for Undergraduate Medical Course Part I, II and III. Available online at: http://www.kimskarad.in/MBBS

[14] Integral University Regulations, Ordinance and Syllabus for MBBS Course in Integral University. Available online at: http://iul.ac.in/IIMSR/MBBSOrdinanceAndSyllabus.pdf

[15] Dr. NTR University Of Health Sciences Hand Book for Students: MBBS Course Regulations. Available online at: http://ntruhs.ap.nic.in/mbbsnavigationframe.html

[16] Rajiv Gandhi University of Health Sciences Undergraduate MBBS Degree Course and Curriculum of Phase I, II and III Subjects. Available online at: http://www.rguhs.ac.in/courses_rguhs.html

[17] Pondicherry University M.B.B.S Syllabus and Regulations (2017–18 onwards). Available online at: http://www.pondiuni.edu.in/sites/default/files/downloads/MBBS.pdf

[18] Jawaharlal Institute of Postgraduate Medical Education and Research MBBS Revised Curriculum Phase I (2017) and MBBS Revised Curriculum Phase II (2018). Available online at: http://www.jipmer.edu.in/sites/default/files/MBBS%20Phase-I%20revised%20curriculum.pdf; http://www.jipmer.edu.in/sites/default/files/Curricular%20reforms%20for%20Phase%20II%20MBBS%20final%2007092018%20%281%29.pdf

[19] Medical Council of India. Available online at: https://www.mciindia.org/CMS/information-desk/for-colleges/ug-curriculum

[20] Tol A Tavassoli E Shariferad GR Shojaeezadeh D. Health-promoting lifestyle and quality of life among undergraduate students at school of health, Isfahan University of Medical Sciences. J Educ Health Promot. (2013) 2:11. 10.4103/2277-9531.108006 24083261 PMC3778574

[21] Musavian AS Pasha A Rahebi SM Roushan ZA Ghanbari A. Health promoting behaviors among adolescents: a cross-sectional study. Nurs Midwifery Stud. (2014) 3:e14560. 10.17795/nmsjournal14560 25414892 PMC4228521

[22] Pati S. Putting tobacco cessation and prevention into undergraduate medical education. Int J Prev Med. (2014) 5:69–75. 24554994 PMC3915476

[23] Raupach T Al-Harbi G McNeill A Bobak A McEwen A Smoking cessation education and training in UK medical schools: a national survey. Nicotine Tob Res. (2014) 17:372–5. 10.1093/ntr/ntu199 25257981 PMC5479510

[24] World Health Organization Tobacco Free Initiative: Code of Practice on Tobacco Control for Health Professional Organizations. Available online at: https://www.who.int/tobacco/wntd/2005/codeofpractice/en/

[25] Warren CW Sinha DN Lee J Lea V Jones NR. Tobacco use, exposure to secondhand smoke, and training on cessation counseling among nursing students: cross-country data from the Global Health Professions Student Survey (GHPSS), 2005–2009. Int J Environ Res Public Health. (2009) 6:2534–49. 10.3390/ijerph6102534 20054453 PMC2790091

[26] Warren CW Jones NR Chauvin J Peruga A GTSS Collaborative Group. Tobacco use and cessation counselling: cross-country. Data from the Global Health Professions Student Survey (GHPSS), 2005-7. Tob Control. (2008) 17:238–47. 10.1136/tc.2007.023895 18474539

[27] Strong A Stoutenberg M Hobson-Powell A Hargreaves M Beeler H Stamatakis E. An evaluation of physical activity training in Australian medical school curricula. J Sci Med Sport. (2017) 20:534–8. 10.1016/j.jsams.2016.10.011 28209318

[28] Swiss Society of Addiction Medicine Substance Use and Addictive Behaviour in the Undergraduate Medical Curriculum in Switzerland: A Teaching Concept. (2016). Available online at: https://www.ssam.ch/d8/sites/default/files/report-pregrad%20training%20addiction%20medicine%20SSAM.pdf

